# Health Literacy in Iranian Women: A Systematic Review and Meta-Analysis

**Published:** 2020-05

**Authors:** Elham CHAROGHCHIAN KHORASANI, Seyedeh Belin TAVAKOLY SANY, Arezoo OROOJI, Gordon FERNS, Nooshin PEYMAN

**Affiliations:** 1. Department of Health Education and Health Promotion, Student Research Committee, School of Health, Mashhad University of Medical Sciences, Mashhad, Iran; 2. Social Determinants of Health Research Center, School of Health, Mashhad University of Medical Sciences, Mashhad, Iran; 3. Department of Epidemiology and Biostatistics, Student Research Committee, School of Health, Mashhad University of Medical Sciences, Mashhad, Iran; 4. Department of Medical Education, Brighton and Sussex Medical School, University of Brighton Falmer Campus, BN1 9PH, UK

**Keywords:** Health literacy, Women, Meta-analysis, Self-efficacy, Self-care behaviors, Iran

## Abstract

**Background:**

Inadequate health literacy (HL) is associated with poorer health outcomes and worse health care. Up to one-half of Iranian women have difficulty in interpreting medical information, and national HL assessment has been limited in Iran. We have undertaken a systematic review of the literature and used a meta-analysis to examine the situation of HL status in Iranian women, and determine the relationship between HL and self-efficacy, and self-care behaviors.

**Methods:**

Six databases (PubMed, Web of Science, Scopus, Google Scholar, Scientific Information Database) and other non-indexed citations were searched using a variety of keywords regarding HL and Iranian women. The bias risk was decreased by the involvement of two independent reviewers assessing study quality and eligibility of included articles.

**Results:**

The average HL scores were in the range of marginal or limited (63.08; 95% CI, 59.83–66.32) in the Iranian women. The HL score was significantly higher among pregnant women (67.55; 95% CI, 32.54–82.57) and was lower in women with chronic disease (57.79; CI, 48.34–67.24). There was a significant association between HL and self-efficacy and self-care behaviors.

**Conclusion:**

The average level of HL in the period of the review was marginal among Iranian women. The relationship of HL with self-efficacy and self-care behaviors was statistically significant but moderate.

## Introduction

Health education experts use the term health literacy (HL) to explain the degree to which people have the “capacity to obtain, process, and understand basic health information and services needed to make appropriate health decisions” (1, 2). HL is a complex issue, and an inadequate HL can negatively affect women’s health, knowledge and her ability to engage in prevention and health behaviors and make informed decisions that will lead to satisfactory health outcomes both for their selves and their family (3, 4). Studies show the effect of inadequate HL on women’s health outcomes. For instance, a recent study on breast cancer risk perceptions of women and HL status (n=5163) reported that women with lower HL demonstrated a less accurate understanding of cancer prevention behaviors than did women with higher health literacy (5). In another study that examined the HL of 529 women residing in Chicago, women with low HL were less likely to have poor cervical cancer screening knowledge (4). In the USA, HL was significantly impacted reproductive healthcare behaviors (e.g., healthy pregnancy, sexual practices, and postpartum behaviors) that are essential to keep women healthy(1, 4). Likewise, in New York, mothers with higher HL skills were more likely to give their child the correct medication based on the drug information sheets and the prescription label (4, 5). Therefore, given the significant burden of HL on the women’s health status, the emphasis has been considered in addressing and evaluating this risk factor to improve health outcomes in different communities.

In Iran, although the epidemiological profile on burden of healthy lifestyle in term of socio-demographic, behavioral, and clinical characteristics have significantly changed (6, 7), the low HL has been increasingly recognized as serious concern for individual’s health (3, 4, 8). To the best of our knowledge, no rigorous and systematic national assessment on HL has been conducted to date examining the overall status of HL in Iranian women. This type of effort to assess HL will be crucial in determining whether the social and health ambitions of Iran for sustainable promotion in the health community are fully achieved (1, 5). Without a synthesis of the current study, it is difficult to set the new stage to clarify and understand the impact of HL on health inequality and health conditions. Given the importance of HL to public health, it would be prudent for the first time to systematically review the previous studies in Iran and determine the situation of HL status among Iranian women, and the relationship between HL and self-efficacy and self-care behaviors.

## Methods

We planned a systematic meta-review in accordance with Preferred Reporting Items for Systematic Reviews and Meta-analyses (PRISMA) guidelines (9) to address the following research question:

What is the health literacy status among Iranian women?
Is there a relationship between self-efficacy and HL among Iranian women?
Is there a relationship between self-care and HL among Iranian women?

### Search Methods

We searched articles until August 6, 2018 from six databases: the PubMed, Web of Science, Scopus, Google scholar, SID, and other non-indexed citations. Further, we hand searched reference lists of all full-text articles to detect additional articles overlooked by the search terms. The search strategy was conducted using the Medical subject heading (MeSH) thesaurus and keywords related to term “health literacy” and combined with the following free terms: “literacy”, “health literacy”, “self-efficacy”, “Iran”, “self-care behaviors” and “women”.

### Data Abstraction and article Screening

Inclusion criteria were as follows: English/Persian language, described the cross-sectional studies conducted in Iranian women, full-text scientific articles published in indexed scientific journals, measured HL using a validated tool and reported on the relationship of HL with socio-demographic characteristics and health outcomes such as self-care behavior, self-efficacy, knowledge, and medication adherence. Articles were excluded if they were: irrelevant results, duplicated results, dissertations, books, and editorial letters. There weren’t restrictions based on age, and date. At this stage, two authors independently screened titles and abstracts of all returned articles to select potentially eligible articles based on inclusion and exclusion criteria. Full-text articles that meet inclusion criteria were downloaded and their results and method sections were evaluated for final inclusion. The two authors were in 100% agreement over the articles included, and a third reviewer resolved any doubts and discrepancies.

### Outcomes

**Fig. 1:**
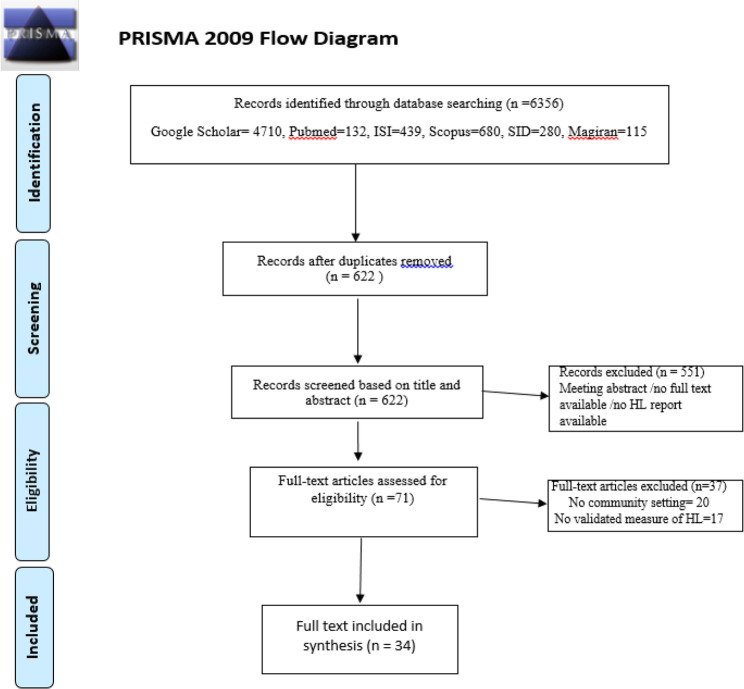
Prisma flow diagram

Overall, 6356 potentially relevant articles were identified. Of these, we selected articles that used a valid specific instrument to measure HL and study protocols were described leading, to the final inclusion of 34 full-text articles for analysis (Fig. 1).

### Data extraction and Quality appraisal

We extracted the following characteristics from all included article: 1) the author(s)/publication year, study design, type of publication, sample size, participant rate and characteristics, inclusion and exclusion criteria, aims; 2) HL score/level of the study population; 3) methodological and instrument approaches used to measure HL and other outcomes; 4) the measure of association between HL and socio-demographic parameters and other outcomes with corresponding *P-*values, for the statistical test. We used the independent dual rating based on the STROBE checklist to evaluate the quality of selected articles. The bias risk was decreased by the involvement of two independent reviewers assessing study quality and eligibility of included articles.

### Meta-analysis

We classified the HL status based on the instruments (TOFHLA, STOFHLA, and HELIA) used in meta-analysis studies. Overall, according to the cut-off points of these instruments, there were three levels of HL interpretation: adequate, marginal/limited, and inadequate. In the meta-analysis, the correlation coefficient (*r*) was also used for examining associations between HL with self-care behaviors and self-efficacy.

Both fixed-effect and random-effect models of effect sizes are shown in the results to compare the results of both models. Likewise, the 95% confidence interval for each outcome measure and their *P*-values were considered. Whereas the fixed-effect model highlighted the homogeneity of the effect size factors and weighted average test, the random-effect model assumes heterogeneity of the factors and un-weighted average test for each outcome. The I-square (I^2^) statistic was used to evaluate the percentage of heterogeneity among studies, which is due to heterogeneity rather than chance. If I^2^ shows the little variation, suggesting a fixed-effects model could be appropriate. The effect size measurement was considered to be significant in this study if the confidence interval (CI) for the effect size was zero. In this study, STATA software, version 14 (Corp LP, USA) was implemented to perform the analysis.

### Ethical approval

The study was conducted after approval from the Mashhad University of Medical Sciences Research Committee (ethical number: IR.MUMS.REC.1397.303).

## Results

Overall, 34 studies including data from 19979 participants, were used in the systematic review and meta-analysis (Table 1, Fig. 1).

**Table 1:** Characterization of the included studies

***References***	***Population***	***Tools***	***Sample size***	***City***	***Average HL***	***Relationship between HL and different variables***		

						***Age***	***Income***	***Education (P-value)***
(33)	Patient	TOFHLA	1120	Saqez	23.2±16.9	r=−0.3 *P*=0.0001		0.0001
(41)			175	Isfahan	63.4±18.01	r=−0.419, *P*‹0.001	R=0.03, *P*=062	-
(34)			212	Shiraz	69.3±18.1	r=−0.146, *P*=0.007		0.000
(35)		HELIA	39	Gorgan	89.2±20	*P*=0.27, r=0.11		0.01
(36)		TOFHLA	72	Yazd	46.6±8.2	*P*>0.05	*P*‹0.001	*P*>0.05
(42)			130	Bushehr	65.2±12.4	*P*=0.0001, r=0.37		0.000
(37)			130	Chenaran	43.7±24.7	*P*=0.451	*P*=0.033	0.417
(38)			251	Tehran	38.9±29.5			*P*‹0.001
(39)			26	Mashhad	53.4±17.6	*P*=0.2		0.012
(40)		HELIA	178	Bastak	95.2±24.6			0.005
(43)			80	Bidgol	109±22.4	*P*‹0.015, R=−0.221		0.001
(10)	Health	TOFHLA	667	5Province	41.1±36.1		*P*=0.0001	0.0001
(12)			562	Kerman	74.6±9.6	*P*=0.231	*P*=0.085	P‹0.001
(13)			240	Izeh	64.04±2.05	*P*‹0.002		P‹0.0001
(14)			250	Roshtkhar	58.7±9.5	*P*=0.02, r=−0.23		0.001
(15)			30	Khaf	55.9±15.3	*P*‹0.05		P‹0.05
(11)			105	Southeast Iran	67.1±16.6	*P*=0.06	*P*=0.01	0.001
(16)		HELIA	659	Tehran	68.3±13.09	*P*=0.0001		0.001
(17)			10436	5 Province	69.02±15.1	*P*‹0.05		P‹0.05
(27)	Pregnant	TOFHLA	250	Bandarabas	70.6±17.2	*P*‹0.001	*P*‹0.001	P‹0.001
(18)	Health	HELIA	330	Lenjan	41.50±9.2	*P*=0.41		0.12
(19)		HELIA	431	Bardaskan	67.6±16.1	*P*=0.009		0.001
(20)			348	Karaj	67.3±14.6			
(21)			204	Tehran	70.5±14.1			
(28)	Pregnant	STOFHLA	400	Urmia	66.04±15.7		*P*=0.01	*P*‹0.001
(29)		HELIA	215	Balochistan	67.6±12.5			
(30)			860	Balochistan	65.9±17.4‘			
(22)	Health	NVS	232	Tabriz	3.3±8.2			
(23)		TOFHLA	120	Mashhad	51.4±12.3	*P*‹0.001	*P*=0.73	*P*‹0.001
(24)		STOFHLA	360	Mashhad	41.3±6.2			
(31)	Pregnant	Maternal	185	Mashhad	42.7±5.6			
(32)		HL	120	Mashhad			*P*=0.008	*P*‹0.001
(25)	Health	HELIA	320	Miyaneh	46.2±0.5			
(26)			242	Tehran				

### Description of Included Studies

#### Study designs and populations

Studies were carried out in 30 different cities in Iran (Table 1). Study populations were community-dwelling healthy women (17 articles from 34 articles (17/34); 50%) (10–26), pregnant women (6/34; 17.64%) (27–32), women with chronic disease (8/34; 23.59%) (33–40), and other disease (3/34; 8.82%) (41–43). Sample sizes ranged from 30 to 10426; five studies had fewer than 100 participants (15, 35, 36, 39, 43) (Table 1).

### Health literacy measurements

Nineteen studies (55.88%) examined HL using the Test of Functional Health Literacy in Adults (TOFHLA) (10–15, 18, 23, 27, 33, 34, 36–39, 41, 42), or Short Test of Functional Health Literacy in Adults (S-TOFHLA) (24, 28), in the translated or culturally adapted version. Twelve studies (35.29%) assessed HL using the Health Literacy for Iranian Adults (HELIA) in the original version (16, 17, 19–21, 25, 26, 29, 30, 35, 40, 43). Three studies assessed HL using the Newest Vital Sign (NVS) and two studies used other health literacy instruments (22, 31, 32) (Table 1).

### Description of Included Results Systematic Review

TOFHLA and HELIA were the most common instruments used to assess HL, while NVS, S-TOFHLA, and REALM were rarely used (Table 1). Overall, 19979 women were included, of whom 6334 women (31.7%) had inadequate HL and another had marginal (8312, 41.6%) and adequate HL (5334, 26.7%). The prevalence rates for low HL ranging from 4.8% to 82.8% (12, 14). Of 20 studies assessed association between age and HL, 12 studies reported a statistically significant inverse association (*P*<0.05) between age and HL (13–17, 19, 23, 27, 33, 34, 42, 43), Likewise, 24/34 studies assess the association between education level and HL (10–19, 23, 27, 28, 32–40, 42, 43), they observed 22/24 (91.6%) significant direct association (*P*<0.05) between education and HL. Eight studies assessed whether the HL affected the level of self-efficacy and self-care behaviors among women population (18, 22–26, 31, 32). The findings showed women with higher HL categories had higher self-efficacy and self-care behaviors.

### Meta-analysis

Of 34 studies included in the systematic review, 27 studies had a design and data, which permitted inclusion in the meta-analysis (10–21, 27–30, 33–43).

### Health literacy status

The total score of 27 individual studies gave an overall effect size (ES) of 63.08 (95% CI: 59.83–66.32) for the average of HL, suggesting that HL score in the range of marginal literacy level among Iranian women. Among surveys using the TOFHLA, S-TOFHLA, and HELIA, the average score of HL were 61.90 (95% CI: 58.46–65.35), 66.04 (95%CI: 35.17–96.91), and 72.70 (95% CI; 62.83–82.56), respectively.

**Fig. 2:**
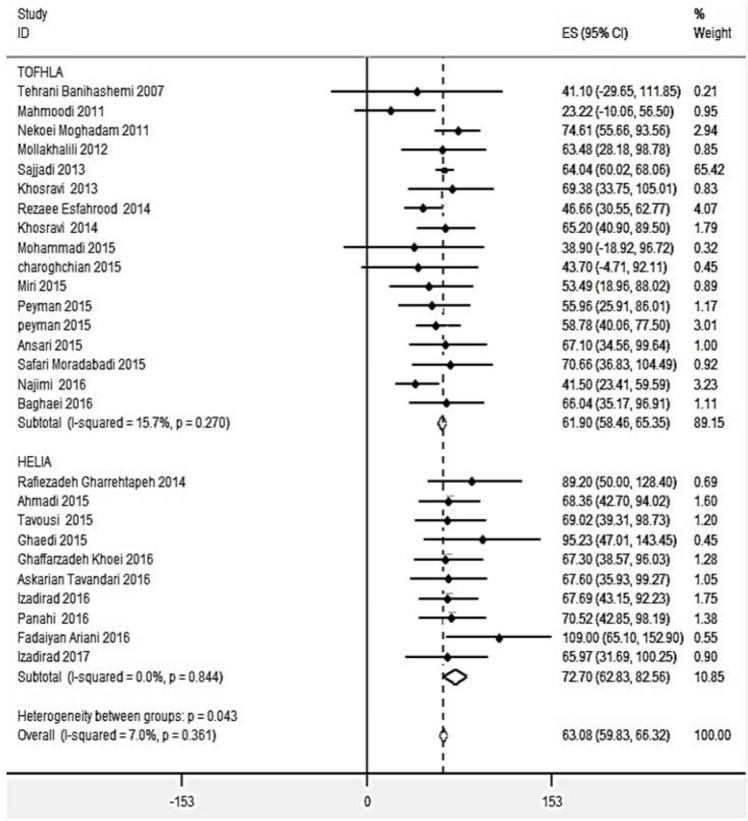
Forest plot of health literacy status, stratified by type of instruments

The I^2^ statistic for overall ES of HL was 7% (*P*=0.361), and it was 15.7% (*P*=0.270) and 0.0% (*P*=0.844) from studies using TOFHLA/STOFHLA and HELIA, respectively. This indicated the homogeneity within the fixed-effects results, therefore, these results were suitable for interpretation (Fig. 2).

The analysis indicated that the use of study setting, age, education of the study population did not have a significant effect on the overall effect size.

The effect measure from studies using HELIA categories was markedly larger than those using TOFHLA and STOHFLA to assess HL (Fig. 2). The individual studies showed that Rafiezadeh, Ghaedi and Fadiayan’s studies (35, 40, 43) were the significant outliers and the overall effect estimate for HELIA scores was 65.75 (marginal category) with the omission of these studies. This result indicated these outliers might affect the pooled effect size for the HELIA score, falling from 89.20 to109.

**Fig. 3:**
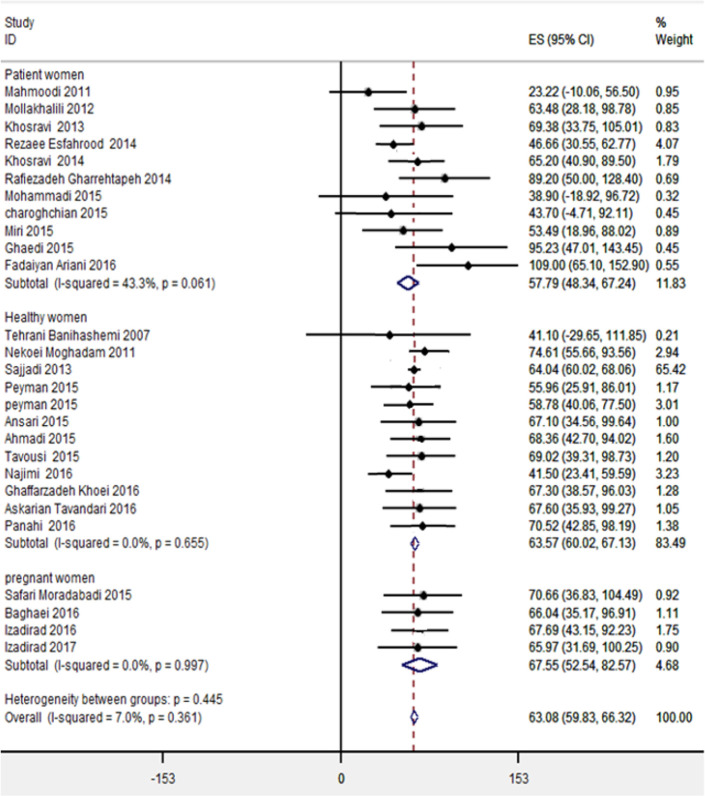
Forest plot of health literacy status, stratified by type of women population

The meta-analysis for study populations showed higher HL scores in pregnant women 67.55 (95% CI; 32.54–82.57), while lower scores were observed in women with other types of disease 57.79 (48.34–67.24) (Fig. 3). Investigation of the individual studies showed that 3 studies (35, 40, 43) were the significant outlier in women with the disease compared to the other studies. The overall effect estimate for this population was 50.3 with the omission of these studies, indicating these outliers may increase the pooled effect size for the overall HL categories among women with a type of disease.

### Association of HL with Self-care and Self-efficacy behaviors

**Fig. 4:**
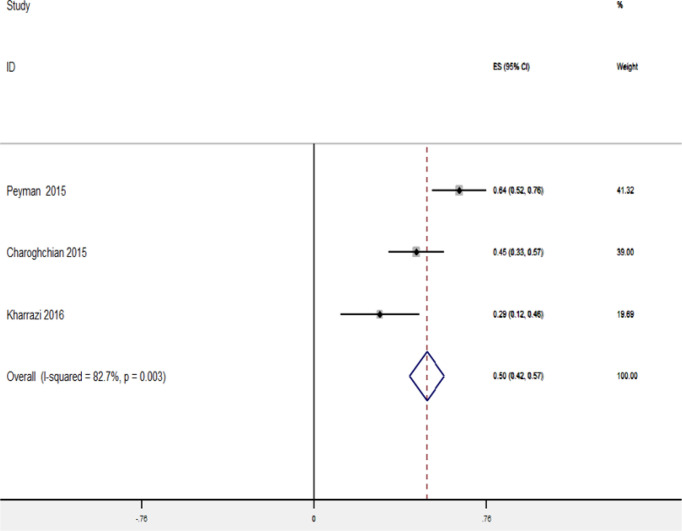
Forest plot of random-effects pooled odds ratios for the association between self-care behaviors and health literacy

Only 8 studies were eligible for assessing the relationship between HL and other domains. The meta-analysis of 5 studies gave an overall fixed-effects R of 0.48 (95% CI: 0.44–0.52) for the association between HL score and self-care behaviors. The I^2^ statistic was 96.1% (*P*<0.001), suggesting that significant heterogeneity within the fixed-effects model, while that data from the random-effects model (r = 0.36; 95% CI: 0.17–0.60, *P*<0.001) were suitable for interpretation (Fig. 4). The Egger’s test (Bias=10.23, *P*=0.393) showed insignificant publication bias.

**Fig. 5:**
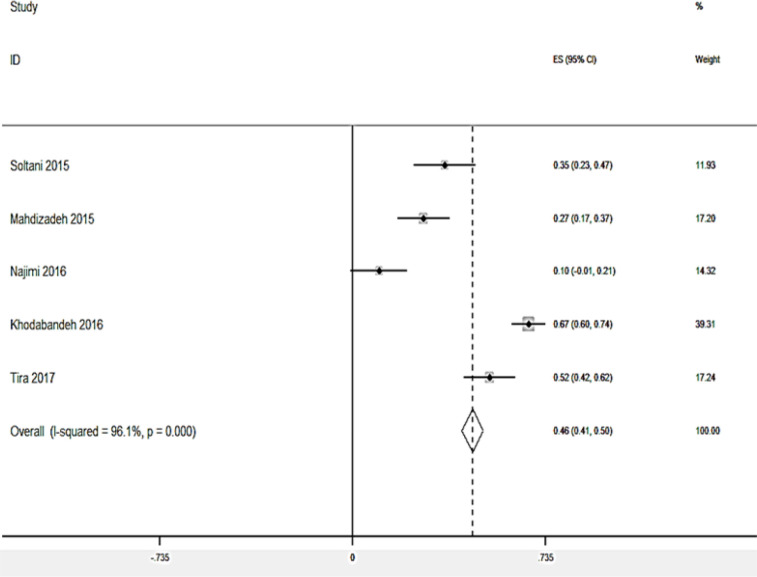
Forest plot of random-effects pooled odds ratios for the association between self-efficacy and health literacy

Within studies examining the association between HL and self-efficacy, fixed-effects R was 0.49 (95% CI: 0.42–0.57), with I^2^ statistic of 82.7%, indicating that heterogeneity was significant. The random-effect R was 0.47 (95% CI: 0.28–0.65). In these analyses, the random and fixed effects Rs were negligibly different; therefore, the fixed-effects R were selected for interpretation because it was consistent and conservative with our result (Fig. 5). The Egger’s test (Bias=17.02, *P*=0.078) showed significant publication bias, while the Trim and Fill test (*P*=0.057) showed insignificant bias. Overall, all studies found a statistically significant direct association between HL and self-care and self-efficacy behaviors.

## Discussion

### Systematic review

Studies included in the systematic review revealed that 31.7% of women had inadequate HL and another 69.1% had adequate or marginal HL skills. The prevalence of inadequate HL in our study ranging from 4.8% to 82.8%. Likewise, our finding showed that TOFHLIA was used more frequently (19/34) than the other questionnaires to assess HL status in Iran. This may due to its representativeness of health-related duties, common usage, and association with fluid cognitive abilities (44).

Differences in HL were found according to age group, education level and income (45, 46). Consistent with the literature, the present study confirms that low HL is associated with low income (45, 46) and income (47). Our findings were also consistent with reviews on the prevalence of HL in the United Kingdom and the USA, which reported older age was significantly associated with having limited HL(44). In our review, older age to be more likely to have limited HL in studies that examined HL as numeracy skills, and reading comprehension by TOFHLA or S-TOFHLA. Older age was also weakly correlated with limited HL in studies that examine HL by psychometric properties, using the HELIA. This result suggests that aging-related HL reduce mainly occurs with abilities requiring fluid cognitive, rather than crystallized cognitive skills. However, the role of cognition in the apparent older age-related HL decline has not been yet well understood and it is not still clear why HL tended to reduce with increasing age (44, 48). Therefore, longitudinal studies are needed to assess the effect of cognition aging on HL decline. In addition, the sample size was generally limited to assess HL- relationship with demographic factors. Thus, difficult to infer whether the impact of these factors could significantly affect HL score, and these results must be interpreted with caution.

### Meta-analysis

The overall HL was marginal in the Iranian women across 27 studies and 18075 women and pregnant women had higher HL score compared to women with chronic disease and healthy women. Our finding showed that adequate HL led to an improvement in self-efficacy and self-care behaviors among women population. Findings in this study are consistent with the 2009 National Assessment of HL in the USA surveyed 19,000 adults showed that a marginal level of HL among women population in the USA (49). They estimated that approximately 36% of the adults in the USA have limited HL skills (4, 5). A recent study by European Health Literacy Survey reported that nearly 50% of all adults in the 8 European countries have marginal (35%) or inadequate (21%) HL skills that negatively affect their life quality (50). In Philadelphia, approximately 50% of the women had low HL levels that were explained as functionally inadequate (4). In Siberia, 44% of women had inadequate HL, and in Taiwan, only 29% of women had marginal or adequate HL (4, 5).

While this meta-analysis reported marginal score as the overall effect of HL in Iranian women, small differences in HL categories within instrument subgroups were observed. Our finding showed that the overall effect of HL measured by HELIA in range of adequate health literacy compared with TOFHLA and STOFHLA that showed marginal effect among women. These differences in the overall score of HL in this study probably due to the difference in the content of instruments examining items range of constructs as “health literacy,” and cutoff scores to defining marginal or adequate HL. For instance, TOFHLA containing comprehension and arithmetic items, which estimated functional skills by the number of correct questions that subject could answer by filling in blank in a written text or answer orally (44, 51). All items in the TOFHLA are extracted from actual health context and real hospital materials such as instructions for health examinations, medical forms and medical prescriptions. But, all items in HELIA are extracted from psychometric characteristics such individual’s perception and attitude (53–55), which examined based on a 5-point Likert scale (52, 53). Such measurement challenges have been previously reported in almost all reviews focusing on HL and make conflicting results (44). These challenges related to the content of instruments and cutoff scores highlight the need to consider the creation of the new HL measures that effectively assess HL skills in different population. However, our finding indicated that some studies overestimated the overall effect estimates of HELIA scores (35, 40, 43). Given the type of population and instrument of the included studies, these finding must be interpreted with caution, and consider the effect of these studies as a significant outlier. While the inclusion of these studies indicated an adequate level of HL among women population, the authors believe that the overall effect estimates with the omission of these studies showed a more accurate effect estimate, indicating the marginal level of HL status, which exactly fits the pattern seen in TOFHLA and STOFHLA.

Differences were also found according to the type of population. The meta-analysis for study populations showed higher HL scores in pregnant women, while the lower score was seen in women with chronic disease. Therefore, the overall effect estimate for the patient’s group may notably decrease the pooled effect size for the overall HL scores among Iranian Women. Although, health disparities have been recently reduced in Iran (54–59), systematic synthesis indicated up to half of the women with chronic disease have trouble using health skills and interpreting medical information (33–40). Pregnant women were more likely to have initiated prenatal care and received preconception counseling with an obstetric provider in health house where women enjoy reproductive health care services free of charge. Furthermore, difference by HL status in Iranian women population could be related to use of information sources that women used to get health information during pregnancy (60, 61). A higher percentage of pregnant women with adequate HL in Iran were more likely to engage in the frequent use of the Internet as a source of health information. Our data appear to support this relationship (60, 61). A study conducted in Japan reported improvement of pregnant women’s HL and its association with quality of prenatal care, access to the media source, community activities to promote the HL and living environment conditions (44, 47).

The meta-analysis indicated that adequate HL led to an improvement in self-efficacy and increased involvement in self-care behaviors among women population. Although the number of studies was limited, it may the meta-analysis lacked enough power to infer whether this association could increase self-efficacy and self-care behaviors, long term, in routine care. Therefore, this finding must be interpreted with caution. Likewise, the effect size of the correlation in our study was weak compared with other determinates of self-efficacy and self-care behaviors such as medication beliefs, disease and cost limitation. Improvement in self-efficacy and self-care behaviors requires myriad determinants and approaches that involve educational, psychological, clinical, financial and behavioral factors, and increasing the HL could be one factor of a multilevel strategy to promote these abilities (44).

Although we made a considerable attempt to use several search strategies and select eligible studies, it is possible that some studies were lost unintentionally. Given the low quality of randomized controlled trial studies on women population in Iran, we decided to include cross-sectional study designs. Furthermore, we can find longitudinal research of HL in Iran. Therefore, the sample size was small and follow-up was generally limited to examine the association of HL with demographic factors and some cognitive parameters. However, these results were not pooled in the meta-analysis and only are interpreted in the systematic review. Likewise, like other research, we cannot exclude the possibility that some studies were missed in their results such as a potential threat to validity and reduced precision. However, our fixed-effects model and random-effects model identified significant homogeneity. The use of the different instruments, study setting, age, education of the study population was not having a significant effect on the measurement of the overall effect. Therefore, our data was suitable for interpretation over the pooled point estimate. We also conducted stratified analysis based on the type of instrument and study population to reduce potential bias. Finally, few studies examined the association between HL and cognitive abilities; therefore, we could not able to report actual conclusions about these associations. However, our findings may guide other studies in the future.

## Conclusion

This review closely adhered guidelines in the PRISMA and Cochrane Handbook for meta-analysiss and systematic review reporting. Based on our findings, it is the first quantitative synthesis of data on Iranian women and HL that systematically examine the HL status and its relationship with health outcomes. This allowed us to examine not only their power of outcome measurement but also how much more likely their score was to be higher or below threshold. Marginal HL is likely to be common among Iranian women and worthy of further research and attention. Higher HL scores were found in pregnant women compared with healthy women and women with other conditions. The theoretical understanding of the status of HL in women population has been hampered by the use of different questionnaires, a lack of longitudinal studies, and few studies assessing cognitive processes. Although the STOFHLA, TOFHLA and, NVS appear to be sensitive to detect HL limitation in the broad functional skills needed to improve health, scoring levels of these instruments need psychometric assessment for comparison against one another. The HELIA appears a promising instrument to assess psychometric characteristics but it less examined in terms of its ability to detect well functional HL skills. In this context, our findings highlighted careful methodological decisions and designing a comprehensive instrument for future studies. A longitudinal study that includes women at different demographic characteristics and cognitive ability are required to promote our understanding of the dynamics of HL variation and its causal processes among women population. In addition, the implications of this review would be practical for public health educators and health promoters to better understand the HL status of women populations as well as variables that affect HL.

## Ethical considerations

Ethical issues (Including plagiarism, informed consent, misconduct, data fabrication and/or falsification, double publication and/or submission, redundancy, etc.) have been completely observed by the authors.
